# Molecular diagnostics and next-generation sequencing reveal real etiological characteristics of invasive *Salmonella* infection in febrile illness in Freetown, Sierra Leone

**DOI:** 10.1080/22221751.2022.2076612

**Published:** 2022-05-23

**Authors:** Jiayong Zhao, Xin Lu, Alie Tie, Esther Ngegba, Lili Wang, Lu Sun, Ying Liang, Michael K. Abdulai, Sununu Bah, Gang Wang, Xiaoping Dong, Doris Harding, Biao Kan

**Affiliations:** a BSL-3 Laboratory, Henan Center for Disease Control and Prevention, Institute of Infectious Disease Prevention and Control, Zhengzhou, People’s Republic of China; b State Key Laboratory of Infectious Disease Prevention and Control, National Institute for Communicable Disease Control and Prevention, Chinese Center for Disease Control and Prevention, Beijing, People’s Republic of China; c Sierra Leone-China Friendship Biological Safety Laboratory, Chinese Center for Disease Control and Prevention, Beijing, People’s Republic of China; d Center for Global Public Health, Chinese Center for Disease Control and Prevention, Beijing, People’s Republic of China; e Chinese Center for Disease Control and Prevention, National Institute for Viral Disease Control and Prevention, Beijing, People’s Republic of China; f Ministry of Health and Sanitation, Freetown, Sierra Leone

**Keywords:** Typhoid fever, blood culture, Widal test, invasive non-typhoidal *Salmonella* infection, next-generation sequencing, metagenomic sequencing

## Abstract

Invasive *Salmonella* infection, which can cause typhoid/paratyphoid fever and invasive non-typhoidal salmonellosis, is a public health burden in Africa. Accurate diagnosis and etiological characterization are required to conduct prevalence and risk estimations for *Salmonella* infection; however, the utilization of optimal techniques and surveillance data are still insufficient. In this study, we performed a laboratory-based survey in Freetown, which is the biggest city in Sierra Leone with a high burden of typhoid fever, by using blood culture and molecular methods but not the Widal test, to estimate the prevalence and aetiology of invasive *Salmonella* infection among fever patients. We found a very low prevalence of typhoid fever in patients with fever during the investigation period, and this prevalence was clearly overestimated by the Widal test. Genome sequencing of the *S*. Typhi isolate from this work revealed that the strain carried multiple antibiotic resistance genes, and an epidemic clone that has existed in West Africa for years was also detected in Sierra Leone. By using metagenomic sequencing, one patient with invasive non-typhoidal salmonellosis was identified as having bacterial co-infections. Our data highlight that *Salmonella* surveillance based on accurate laboratory diagnosis and genome sequencing needs to be strengthened to provide a better estimation of the real epidemics and enable potential risk assessment by etiological analysis in Africa. Even in a laboratory with only basic equipment, it is possible to conduct next-generation sequencing for pathogen discovery in bloodstream infections and to determine the etiological characteristics of pathogene without complex combinations of laboratory methods.

## Introduction

Invasive *Salmonella* infection, presenting as typhoid or paratyphoid fever or as invasive non-typhoidal salmonellosis, causes a significant burden of disease in sub-Saharan Africa [1]. Typhoid and paratyphoid fever are acute systemic diseases, and some severe cases can be fatal. Outbreaks and epidemics of typhoid and paratyphoid fever are public health problems worldwide, especially in regions lacking access to safe water and food [2–4]. About 10.9 million cases of typhoid fever and 116,800 deaths were attributed to typhoid globally in 2017, with 15.9% of deaths occurring in sub-Saharan Africa [5]. Epidemics of typhoid and paratyphoid fever in Africa are less well understood compared with those in other parts of the world, largely because of the limited access to accurate laboratory diagnostic methods and lack of pathogen genome sequencing data there. Attempts to estimate the disease burden of typhoid fever from published studies found that information was too scarce to estimate anything other than a crude incidence rate (50 cases per 100,000 individuals) in a population of approximately 820 million people [6]. The 2010 Global Burden of Disease study estimated similar incidence rates for typhoid fever [7], but another study [8] reported higher estimates (724.6 cases per 100,000 individuals) following the incorporation of data from a study in Kenya [9]. In Malawi and South Africa, typhoid fever affects mainly children aged 5–15 years [10], and in urban areas of Kenya, crude incidence rates of typhoid fever can reach 247 cases per 100,000 individuals [11].

An estimated 535,000 cases of invasive non-typhoidal salmonellosis and 77,500 associated deaths occurred globally in 2017, with the highest disease incidence (34.5 cases per 100,000 person-years) observed in sub-Saharan Africa [12]. In a meta-analysis of community-acquired bloodstream infections among hospitalized patients, non-typhoidal *Salmonella* was the most common isolated pathogen followed by *Escherichia coli* [13]. In Thailand, a population-based bloodstream infection surveillance detected more than 10 pathogens, with non-typhoidal *Salmonella* (NTS) ranking fifth most common [14].

Infections caused by many different pathogens can present with fever as the major symptom. The Widal test is still used as a common method for the diagnosis of typhoid fever in clinical laboratories in developing regions, despite its low specificity and low sensitivity for the diagnosis of typhoid and paratyphoid fever [3, 15]. Disease surveillance and burden estimates based on Widal test results may lead to misunderstanding of the epidemiology of typhoid and paratyphoid fever in some regions, such as Nigeria [3], Egypt [16], and Pakistan [17].

In Sierra Leone, located in West sub-Saharan Africa, large numbers of typhoid and paratyphoid fever cases are reported each year. The incidence of typhoid fever in Sierra Leone was estimated as 194–925 cases/100,000 person-years [2]; all cases used to produce this estimate were diagnosed from Widal test results and/or clinical manifestations. Etiological diagnosis for typhoid and paratyphoid fever is lacking, and the consequent reliance on less accurate diagnostic methods may result in an incorrect estimation of the typhoid/paratyphoid fever burden.

Here, we performed a clinic-based study and etiological survey of invasive *Salmonella* infection among patients with fever in Freetown, the biggest city in Sierra Leone. Our goal was to re-evaluate the prevalence of invasive *Salmonella* infection in Freetown from etiological evidence.

## Materials and methods

### Study patients and inclusion criteria

**Figure 1.**
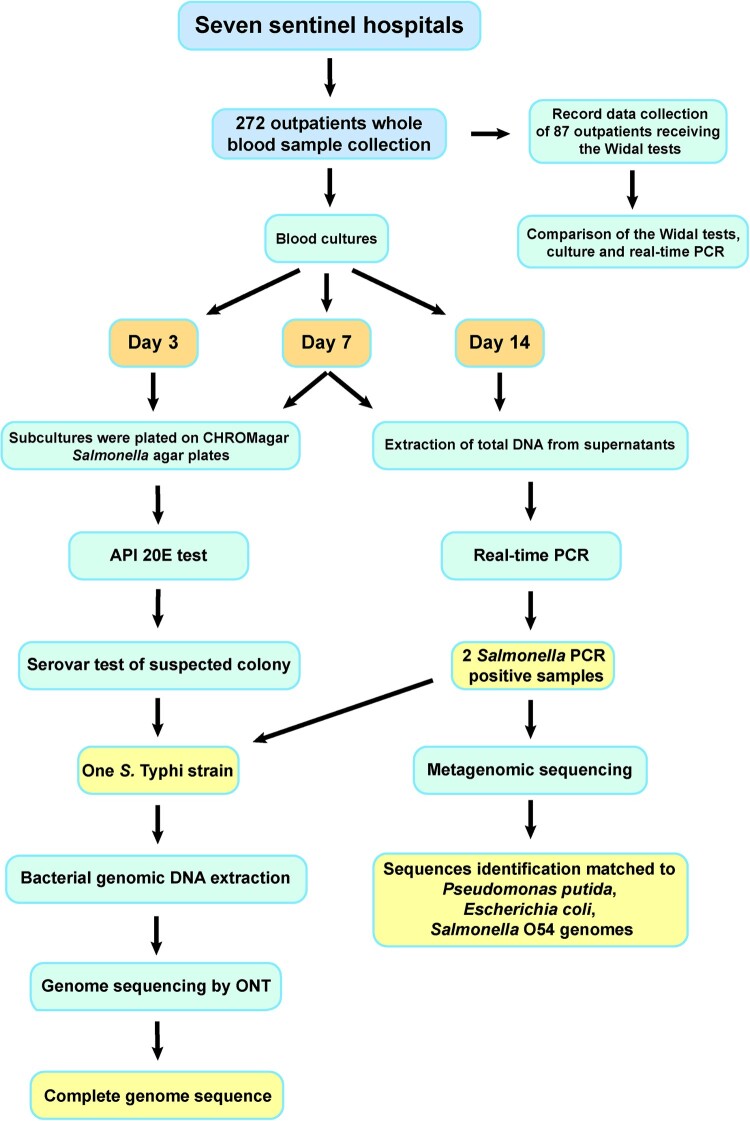
Laboratory procedure and results from examining patient blood samples.

To investigate the epidemiology of fever caused by invasive *Salmonella* infection, an etiological survey of patients with suspected or clinically diagnosed fever was performed as part of the “Etiological Surveillance of Typhoid and Paratyphoid fever in Western, Sierra Leone” study, which was conducted by Chinese Centre for Disease Control and Prevention and Ministry of Health and Sanitation of Sierra Leone and belonged to the Sierra Leone – China Second Phase of the Fixed Biological Safety Laboratory Technical Cooperation Project; the study protocol for that project was reviewed by the National Laboratory and Blood Safety Services of the Ministry of Health and Sanitation, Sierra Leone. Surveillance sites included seven sentinel hospitals (Lumley Hospital, 34 Military Hospital, Waterloo Hospital, Connaught Hospital, Sierra Leone-China Friendship Hospital, Rokupa Hospital, and Emergency Hospital) in the west zone of Freetown from April to June 2019 (Figure 1). Outpatients with body temperatures of >37 °C for more than two days were enrolled. Basic clinical data, including patient age, gender, occupation, and fever onset date, were collected. A whole blood sample (8–10 mL/sample) was collected from each outpatient.

### Bacterial culture, identification, and real-time PCR

Blood cultures were performed by aseptically introducing 8 mL of blood into biphasic blood culture bottles. The cultures were incubated for 14 days at 37 °C. On day 3 and day 7, subcultures were plated on CHROMagar *Salmonella* agar plates (CHROMagar, Paris, France). Suspected *Salmonella* isolates were identified using API 20E test strips (bioMérieux, Marcy-l'Étoile, France).

*Salmonella* isolates were plated on Columbia nutrient agar plates (Oxoid, Basingstoke, UK) and cultured overnight (16–18 h) at 37 °C. The isolates were characterized by agglutination of the O antigen, H1 phase antigen, and H2 phase antigen; normal saline was used as a self-agglutination control. Antigenic properties were confirmed in accordance with the Kauffmann–White serotyping table.

Total genomic DNA was extracted from the supernatants of 7-day and 14-day blood cultures and screened for the presence of *Salmonella* by using real-time PCR detection kits (Liferiver, Shanghai, China).

### Bacterial genomic DNA extraction and metagenomic sequencing

*Salmonella* isolates were streaked on Luria–Bertani (LB) [18] agar plates and incubated at 37°C for 18 h. A single colony was subcultured overnight at 37°C in LB broth. DNA was extracted using the Wizard Genomic DNA Extraction Kit (Promega, Madison, WI, USA). An Oxford Nanopore Technologies (ONT; Oxford, UK) sequencing library was constructed using the field sequencing kit (SQK-LRK001), and sequencing was carried out on a MinION device using flow cell type R9.4 (FLO-MIN106D). Porechop (https://github.com/rrwick/Porechop) was used to trim adaptor sequences from raw nanopore sequencing reads and for demultiplexing. Short reads of <2 kb in length were filtered for further analysis. Genome assembly was conducted using SPAdes.

Total genomic DNA was extracted from culture supernatants of *Salmonella* PCR-positive samples using the QIAamp DNA Mini Kit in accordance with the manufacturer’s protocols. An ONT sequencing library was constructed using the field sequencing kit (SQK-LRK001), and sequencing was carried out on a MinION device using flow cell type R9.4 (FLO-MIN106D).

To reveal the genetic relationship among the *S*. Typhi isolate obtained in this study and other *S*. Typhi strains, genomic sequences of *S*. Typhi strains were retrieved from GenBank and used to construct a maximum-likelihood (ML) tree, with *S*. Paratyphi A (GCA000026565), *S*. Typhimurium (GCA000006945), and *S*. Enteritidis (GCA000750395) used as outgroups. The coding sequences from the strains were grouped together, and a non-redundant homologous gene set was computed for the sequences using CD-HIT. We searched the homologous genes in the non-redundant homologous gene set for the coding sequences of each strain using BLAST+. If the homologous gene for a gene in the non-redundant homologous gene set existed in all selected strains and had just one copy per strain, the gene was considered a core gene. The core genes were then aligned and merged, and IQ-TREE was used to construct the ML tree.

Drug-resistance gene content and plasmid replicon type were analysed *in silico* using online tools (http://www.genomicepidemiology.org/). Prophage regions were identified using PHASTER (http://phaster.ca/).

For metagenomic identification of pathogens, data were processed using Kraken2, the MGI Pathogeny Fast Identification pipeline, and the MicroFuture Pathogen Identification pipeline. Bowtie2 (http://bowtie-bio.sourceforge.net/bowtie2) and SAMtools (https://www.htslib.org/) were used to extract *Salmonella* sequence reads.

## Results

### Patient characteristics and Widal test results

Blood samples were collected from 272 patients who had at least a mild fever (body temperature of >37 °C) (Table 1). Most cases had moderate or high body temperatures of >38 °C. The interval between fever onset and hospital visit was less than five days in 68.8% of patients, 6–10 days in 9.2% of patients, and 11 days or longer in 12.1% of patients. The major symptoms were headache (83.8%, 228/272), and chill (51.1%, 139/272). The majority of study participants were students (34.9%, 95/272) or workers (14.7%, 40/272), and 61.8% (168/272) of the study population was aged 19–50 years old.

**Table 1.** Characteristics of the patients enrolled in this study.

Body temperature (°C), n (%)	37-37.5	37.5-38	38–39	39–40	>40								
Total	53 (19.49%)	40 (14.71%)	122 (44.85%)	54 (19.85%)	3 (1.10%)								
Widal test	28 (32.18%)	9 (10.34%)	26 (29.89%)	21 (24.14%)	3 (3.45%)								
Age (years), n (%)	<5	6–18	19–30	31–50	51–60	>60	unknown						
Total	16 (5.88%)	45 (16.54%)	86 (31.62%)	82 (30.15%)	15 (5.51%)	10 (3.68%)	18 (6.62%)						
Widal test	11 (12.64%)	19 (21.84%)	26 (29.89%)	25 (28.74%)	4 (4.60%)	1 (1.15%)	1 (1.15%)						
Occupations, n (%)	student	worker	housekeeper	trader	farmer	medical worker	teacher	fisherman	food maker	driver	police	sporadic children	others
Total	95 (34.93%)	40 (14.71%)	25 (9.19%)	20 (7.35%)	13 (4.78%)	11 (4.04%)	11 (4.04%)	9 (3.31%)	5 (1.84%)	2 (0.74%)	2 (0.74%)	2 (0.74%)	37 (13.60%)
Widal test	44 (50.57%)	14 (16.09%)	7 (8.05%)	0 (0%)	1 (1.15%)	5 (5.75%)	1 (1.15%)	2 (2.30%)	4 (4.60%)	0 (0%)	2 (2.30%)	0 (0%)	7 (8.05%)
Symptoms, n (%)	headache	chill	bellyache	jaundice	arthralgia	oliguria or anuria or gross	eyelid edema	diarrhea	relative infrequent pulse	purpura	petechiae or ecchymosi		
Total	228 (83.82%)	139 (51.10%)	81 (29.78%)	64 (23.53%)	23 (8.46%)	18 (6.62%)	9 (3.31%)	9 (3.31%)	6 (2.21%)	6 (2.21%)	1 (0.37%)		
Widal test	66 (75.86%)	36 (41.38%)	21 (24.14%)	18 (20.69%)	21 (24.14%)	14 (16.09%)	7 (8.05%)	4 (4.60%)	4 (4.60%)	0 (0%)	1 (1.15%)		

Widal test results were available for 87 patients (42 female patients, 41 male patients and four patients of unknown sex); 37, 26, and 24 of these patients had low, moderate (38-39 °C), and high body temperatures (>39 °C), respectively. Among the 87 patients with available Widal test results, 78 patients (90.0%) had positive Widal test results (Table 1 and Figure 1). The high rate of positive Widal test results could indicate a significant prevalence of typhoid fever in Freetown.

### Bacterial culture and real-time PCR detection of *Salmonella* infection

In plate cultures of samples from blood culture bottles, only one *S*. Typhi isolate (case number: WGH-01) was obtained. This isolate was characterized using the API 20E test and serotyping. Overall, the blood culture results indicate that only 0.4% of the patients with fevers were positive for *Salmonella* infection. The patient from whom the isolate was obtained was not tested by the Widal method.

In parallel, DNA was extracted from the supernatants of all 272 blood culture bottles after 7 and 14 days of incubation and subjected to real-time PCR for *Salmonella* detection. Two (0.7%) *Salmonella*-positive samples were identified. One of these *Salmonella*-positive samples was from case WGH-01; this is the same case that provided the sample from which the *S*. Typhi isolate mentioned above was obtained. For the other PCR-positive sample (case number: LH:19-056), colonies were cultured, but no *Salmonella* strains were identified. Among the 87 patients who were tested via the Widal method, none were both culture positive and real-time PCR positive for *Salmonella*.

### Metagenomic sequencing of blood culture

Metagenomic sequencing was carried out on the *Salmonella*-PCR positive sample from case LH:19-056. DNA was extracted from the supernatant of the blood culture bottle from this case after it had been incubated for 14 days and was sequenced using a MinION instrument. About 3.2 Gb of sequencing data (2,912,377 reads) were obtained after 48 h of sequencing. The maximum read length was approximately 75.1 kb (79,201 reads of ≥2 kb in length; 30,582 reads of ≥5 kb in length; and 8,878 reads of ≥10 kb in length). Metagenomic sequencing yielded a data set of 2,872,916 clean reads, of which 2,253 reads corresponded to *Salmonella* (relative abundance: 0.3%). A comparison of *Salmonella* reads with the *Salmonella* determinants database revealed that the isolate in this sample harboured a O:54 lipopolysaccharide O-antigen. Because neither *S*. Typhi nor *S*. Paratyphi serovars express the O:54 antigen, non-typhoidal *Salmonella* may have caused the bloodstream infection in this case.

**Figure 2.**
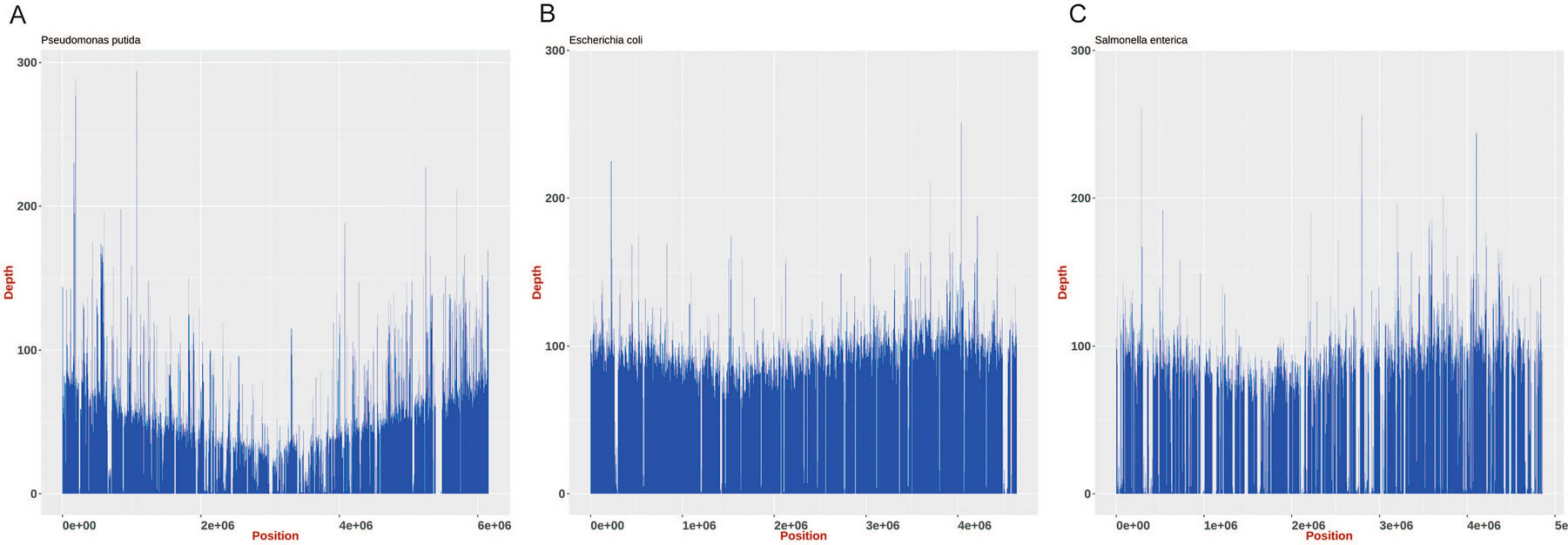
Mapping of genome assemblies for *P. putida, E. coli,* and *Salmonella* generated from ONT MinION reads to the respective reference genomes (*P. putida*: NC_021505.1, *E. coli*: NC_000913.3, and *Salmonella*: NC_003197.2). The mean sequencing coverage for the *P. putida*, *E. coli,* and *Salmonella* genomes assembled in this study was approximately 39.8%, 80.1%, and 57.1%, respectively.

Reads related to *Pseudomonas putida* (relative abundance: 41.1%) and *E. coli* (relative abundance: 33.9%) were observed in the sequencing data for this sample. These reads were mapped to the reference genomes of *P. putida* (NC_021505.1), *E. coli* (NC_000913.3), and *S.* Typhimurium (NC_003197.2); approximately 85.4%, 96.3%, and 78.8% coverage, respectively, was obtained (Figure 2). Several antimicrobial resistance genes were identified, including *aph(3'’)-Ib*, *aph(6)-Id*, *dfrA14*, *qnrS4*, *mdt*, and *bla*_TEM_. Thus, this isolate may be resistant to aminoglycosides, diaminopyrimidines, fluoroquinolones, antimicrobial peptides, and cephalosporins.

### Genome sequencing of a *S*. Typhi strain isolated from blood

**Figure 3.**
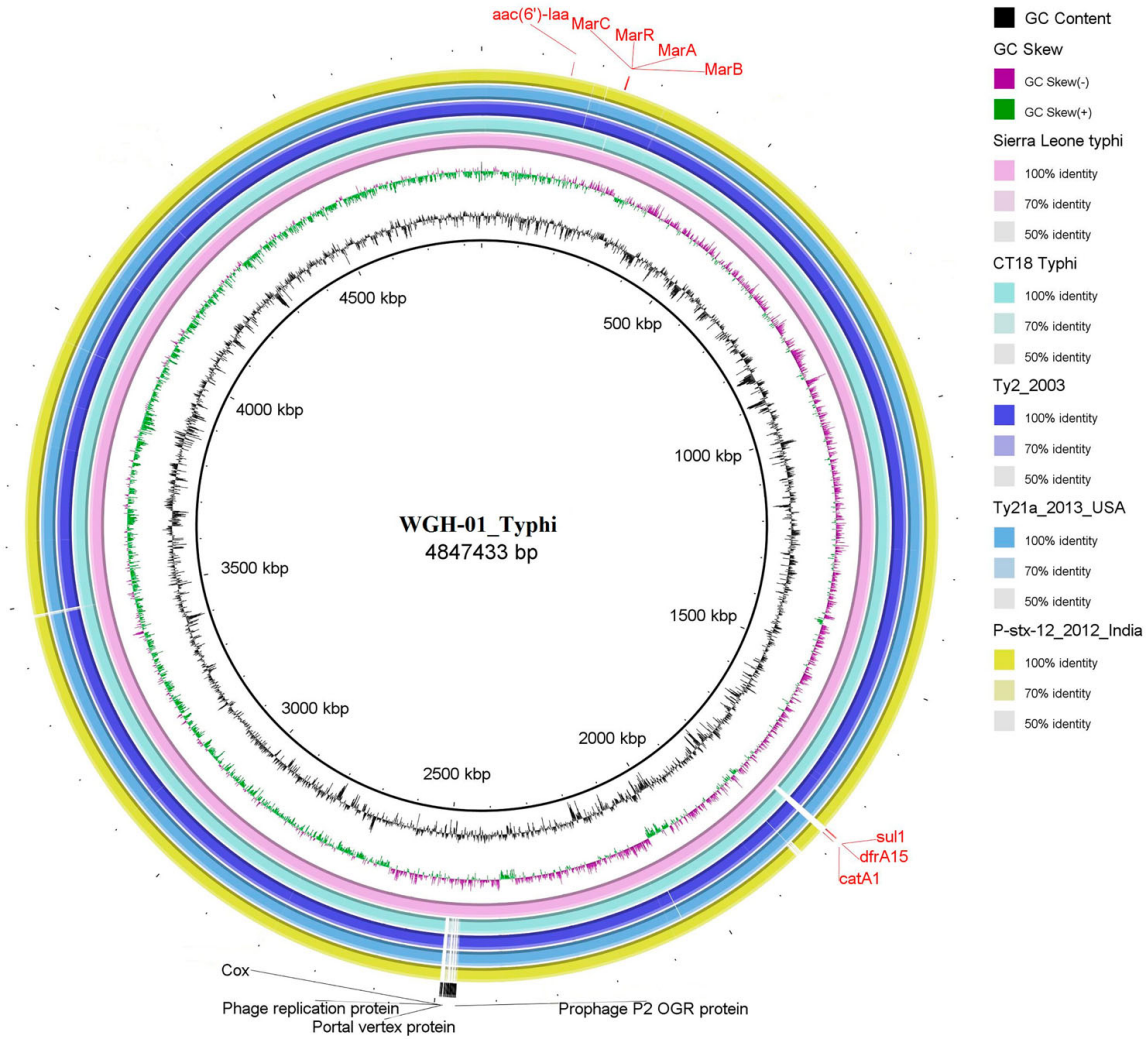
Comparison of the genome sequence of the WGH-01_Typhi isolate obtained in Sierra Leone with other *S*. Typhi complete genome sequences.

Genomic DNA was extracted from isolate WGH-01 and sequenced for 48 h using a MinION instrument. Approximately 2 Gb of sequencing data (188,674 reads) was obtained. The maximum read length was approximately 190.7 kb. The genome was assembled using SPAdes, and one circular chromosome of 4,847,433 bp (GC content: 52.3%) was obtained (GenBank accession number) (Figure 3). A total of 4,593 protein-coding genes and 74 tRNAs were predicted to be present in the assembled genome. Analysis using SeqSero-1.2 showed that isolate WGH-01 belonged to serovar Typhi (9:d:-). By searching ResFinder with the assembled genome sequence, four antimicrobial resistance genes were identified: *aac(6’)-Iaa*, *catA1*, *dfrA15,* and *sul1*, corresponding to aminoglycoside, phenicol, trimethoprim, and sulfonamide resistance, respectively. Among these resistance genes, *catA1*, *dfrA15*, and *sul1* were found within a Tn3 family transposon. We also analysed the chromosomal point mutations for antibiotic resistance. The site mutations of E84K in *parC* and A67P in *gyrA* were found; these mutations are known to confer fluoroquinolone resistance, suggesting that isolate WGH-01 may be resistant to fluoroquinolone. In addition, 17 prophage regions were identified; four regions were intact, nine regions were incomplete, and four regions had questionable integrity.

**Figure 4.**
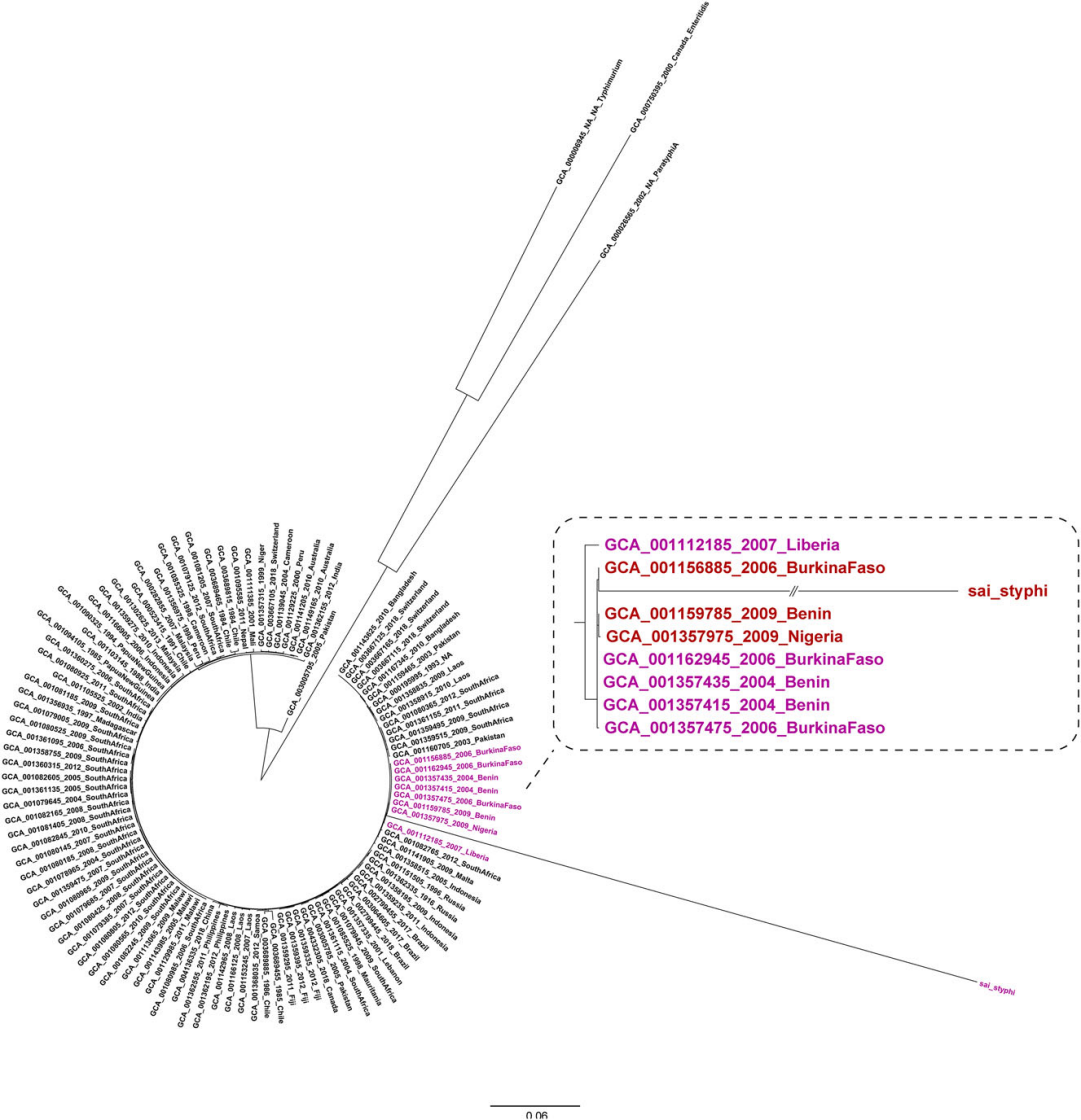
Phylogenetic analysis of the *S*. Typhi isolate obtained in the present study conducted with sequences from GenBank. The phylogenetic analysis was conducted on the *S*. Typhi isolate from our study outpatients and on another 105 *S*. Typhi sequences retrieved from GenBank. The genomes of *S*. Paratyphi A (GCA000026565), *S*. Typhimurium (GCA000006945), and *S*. Enteritidis (GCA000750395) were used as outgroups.

To reveal the genetic relationship of the *S*. Typhi isolate obtained in this study with other epidemic strains, 105 *S*. Typhi genomic sequences from different countries were retrieved from GenBank. *S*. Paratyphi A (GCA000026565), *S*. Typhimurium (GCA000006945), and *S*. Enteritidis (GCA000750395) were used as the outgroups to construct a ML tree. In this ML tree, the *S*. Typhi isolate obtained in our study was clustered with *S*. Typhi strains from Nigeria, Benin, Burkina Faso, and Liberia (Figure 4), which are countries in West Africa and near Sierra Leone. Most of these *S*. Typhi strains were isolated in years from 2004–2009, which strongly suggests that clonal transmission of *S*. Typhi has been occurring within this region for years.

## Discussion

Accurate diagnosis of typhoid/paratyphoid fever is required for optimal patient treatment, detection of outbreaks and epidemics, quantitation of disease burden, and implementation of health interventions. Because of the nonspecific clinical features of *S*. Typhi and *S*. Paratyphi infections, typhoid/paratyphoid fever is often misdiagnosed, especially in low-income regions with limited access to sophisticated laboratory techniques. In this study, we estimated the prevalence of typhoid fever in Freetown, Sierra Leone, where typhoid fever is considered a common disease. Only 0.4% of study patients had their typhoid fever diagnosis confirmed by bacterial culture and molecular diagnosis results, suggesting that this disease may be over-diagnosed and that its prevalence may be overestimated owing to the unreliable data from available clinical and accessory laboratory methods.

The Typhoid Fever Surveillance in Africa Programme has firmly positioned typhoid fever as a public health priority in sub-Saharan Africa [19]. However, because of limited access to culture-based laboratory methods in many countries in Africa, the diagnosis of typhoid/paratyphoid fever in these locations relies mainly on clinical symptoms or Widal test results. Despite the Widal test having several advantages, e.g. it is relatively affordable, easy to perform, and requires minimal equipment or expertise, its diagnostic accuracy is low [20]. Using blood culture results as the gold standard, the sensitivity and specificity of the Widal test were reported to be 34.1% and 42.8%, respectively [21]. Laboratory diagnosis of typhoid fever is largely dependent on the detection of organisms in the blood by PCR or culture methods [22]. In the present study using blood culture and real-time PCR to diagnose *S*. Typhi infection, only one patient with a clear etiological diagnosis of *S*. Typhi infection was confirmed among 272 patients with fever. This finding suggests that the incidence of typhoid fever in Freetown during the study period was quite low. In addition, no *S*. Typhi was detected in any of the 78 Widal test-positive blood samples, confirming previous reports that positive Widal tests have low predictive value for typhoid fever.

In addition, the Widal test has cross-reactivity with other pathogens, yielding positive results for patients infected with other *Salmonella* species or those with malaria. One study reported that 48.3% of febrile patients with malaria parasitemia had false-positive Widal tests owing to antigenic cross-reactivity [3]. Sierra Leone has one of the highest malaria burdens according to a report from World Health Organization (WHO) [23]. Hence, the Widal test should not be used as the primary method for typhoid fever diagnosis in regions with endemic or epidemic malaria. Blood culture and molecular detection methods for diagnosing typhoid fever require significant laboratory infrastructure. Although these techniques are not widely available in Sierra Leone at present, capacity building should be prioritized, including starting culture and PCR reference laboratories in public health departments and large hospitals. These capacities can be extended to other units in stepwise approach.

Invasive non-typhoidal *Salmonella* is a major public health concern. In this study, we identified blood infections and co-infections by non-typhoidal *Salmonella*. In Africa, the increased incidence of invasive non-typhoidal salmonellosis has confounded estimates of the typhoid fever burden [24, 25]. Because of the lack of confirmatory methods for *Salmonella spp*. isolation and serotyping, enteric fever caused by *S*. Typhi or *S.* Paratyphi A cannot be discriminated from non-typhoidal salmonellosis. A patient with invasive non-typhoidal *Salmonella* and *P. putida* co-infection was identified in our study. This illustrates the demand for clear etiological discrimination in patients with fever. *P. putida* is widely found on hospital surfaces and moist environments and, as an opportunistic pathogen, can cause bloodstream infections [26, 27]. Therefore, bloodstream infection surveillance is essential to characterize the public health threat [14].

The co-existence of non-typhoidal *Salmonella* with other bacteria in the blood of one patient from this study was demonstrated by metagenomic sequencing. Although no *Salmonella* isolates were obtained by blood culture from this case, we excluded infection by *S*. Typhi or *S*. Paratyphi because the sequencing results showed that only genes encoding the O:54 antigen were present. The relative abundance of sequencing reads related to *Salmonella* was 100 times lower compared with those of sequencing reads related to *P. putida* and *E. coli*. Therefore, *Salmonella* colonies growing from blood culture in this case may have been relatively rare and thus difficult to pick. In the absence of sequencing technologies, multiplexed diagnostic tests, and laboratory culture equipment, the pathogens responsible for coinfections may be hard to detect. Metagenomic next-generation sequencing enables the detection of nearly all known pathogens simultaneously [28]. Nanopore sequencing has also been previously applied in field laboratories, including during the 2014–2016 Ebola outbreak in West Africa [29] and during the Zika and Yellow fever outbreaks in Brazil [30, 31]. Our study demonstrates that Nanopore sequencing is practical for etiological identification of blood infections or other severe infections and can performed in field laboratories with limited infrastructure.

In addition to using blood culture and molecular methods to obtain an accurate diagnosis, we also performed genome sequencing on the *S*. Typhi isolate from this study. A definite cluster was found containing our isolate and *S*. Typhi strains isolated from cases in other West African countries in previous years. Our isolate confirms that this epidemic clone also exists in Sierra Leone. Thus, genome sequencing can provide a warning signal regarding the existence of an epidemic clone, and our finding highlights the necessary of conducting genomic surveillance of pathogens in Africa.

In summary, this study found that the prevalence of typhoid fever in Freetown may be overestimated when using the Widal test result as the primary criterion for diagnosis. Training should be provided for clinicians to increase awareness that the Widal test does not provide a reliable diagnosis of typhoid fever, especially in regions with high malaria rates. This study does not exclude the potential for epidemics of enteric fever in Sierra Leone. On the contrary, our finding of a patient with typhoid fever, which belongs to a cluster of epidemic clones that appeared in some countries near Sierra Leone in West Africa and also carries some antibiotic-resistance genes, may be an alarm for the risk of typhoid fever spread and outbreak, especially in regions with poor sanitation. Laboratory-based confirmation methods for enteric fever diagnosis are needed in African countries to establish capacity in public health and clinical laboratories with reference functions. This would promote the ability of these techniques to enable accurate diagnosis, discriminate between blood infections by non-typhoidal *Salmonella* and other pathogens, and enhance outbreak detection and disease burden estimation. Pathogen genome sequencing and metagenomic sequencing are feasible methods for pathogen monitoring in Africa.

## Data Availability

The datasets used and/or analysed during the current study available from the corresponding author on reasonable request.
